# Neuroprotective Effects of Annexin A1 Tripeptide after Deep Hypothermic Circulatory Arrest in Rats

**DOI:** 10.3389/fimmu.2017.01050

**Published:** 2017-08-30

**Authors:** Zhiquan Zhang, Qing Ma, Bijal Shah, G. Burkhard Mackensen, Donald C. Lo, Joseph P. Mathew, Mihai V. Podgoreanu, Niccolò Terrando

**Affiliations:** ^1^Department of Anesthesiology, Duke University Medical Center, Durham, NC, United States; ^2^Center for Drug Discovery, Department of Neurobiology, Duke University Medical Center, Durham, NC, United States; ^3^Department of Anesthesiology & Pain Medicine, University of Washington Medical Center, Seattle, WA, United States; ^4^Center for Translational Pain Medicine, Duke University Medical Center, Durham, NC, United States

**Keywords:** memory, neuroinflammation, NF-κB, postoperative cognitive dysfunction, resolution of inflammation, surgery

## Abstract

Resolution agonists, including lipid mediators and peptides such as annexin A1 (ANXA1), are providing novel approaches to treat inflammatory conditions. Surgical trauma exerts a significant burden on the immune system that can affect and impair multiple organs. Perioperative cerebral injury after cardiac surgery is associated with significant adverse neurological outcomes such as delirium and postoperative cognitive dysfunction. Using a clinically relevant rat model of cardiopulmonary bypass (CPB) with deep hypothermic circulatory arrest (DHCA), we tested the pro-resolving effects of a novel bioactive ANXA1 tripeptide (ANXA1sp) on neuroinflammation and cognition. Male rats underwent 2 h CPB with 1 h DHCA at 18°C, and received vehicle or ANXA1sp followed by timed reperfusion up to postoperative day 7. Immortalized murine microglial cell line BV2 were treated with vehicle or ANXA1sp and subjected to 2 h oxygen–glucose deprivation followed by timed reoxygenation. Microglial activation, cell death, neuroinflammation, and NF-κB activation were assessed in tissue samples and cell cultures. Rats exposed to CPB and DHCA had evident neuroinflammation in various brain areas. However, in ANXA1sp-treated rats, microglial activation and cell death (apoptosis and necrosis) were reduced at 24 h and 7 days after surgery. This was associated with a reduction in key pro-inflammatory cytokines due to inhibition of NF-κB activation in the brain and systemically. Treated rats also had improved neurologic scores and shorter latency in the Morris water maze. In BV2 cells treated with ANXA1sp, similar protective effects were observed including decreased pro-inflammatory cytokines and cell death. Notably, we also found increased expression of ANXA1, which binds to NF-κB p65 and thereby inhibits its transcriptional activity. Our findings provide evidence that treatment with a novel pro-resolving ANXA1 tripeptide is neuroprotective after cardiac surgery in rats by attenuating neuroinflammation and may prevent postoperative neurologic complications.

## Introduction

Resolution of inflammation was once believed to be a passive process, but we now know that it involves a cascade of coordinated events that is initiated as inflammation begins (1, 2). Defective resolution and non-resolving inflammation contribute to a chronic and maladaptive state that characterizes several diseases ranging from atherosclerosis to rheumatoid arthritis (3). Endogenous mediators, including lipids biosynthesized from omega-3 fatty acids, gases such as carbon monoxide, and certain proteins, promote resolution of inflammation, and restore homeostasis without causing unwanted side effects by optimizing the body’s natural chemistry to safely regulate inflammatory molecules (4). Indeed, novel treatment strategies for inflammatory conditions use “resolution agonists” to modulate and enhance these endogenous mediators and signaling pathways (5, 6).

Annexin A1 (ANXA1), a 37-kDa glucocorticoid-regulated protein, is an exemplary resolution agonist that signals through the G protein-coupled receptors FPR2/ALX and FPR1 to regulate calcium influx into the cell (7). ANXA1 also exerts potent anti-inflammatory actions by regulating leukocyte diapedesis, efferocytosis, and pro-inflammatory mediators following infection or injury (8, 9).

Strategies to activate these endogenous inflammation “stop signals” are gaining considerable interest (10). This innovative line of research has led to ANXA1 peptidomimetics, which are designed to boost activation of naturally occurring pro-resolving and anti-inflammatory mechanisms (11). Perretti and colleagues first developed a peptide that is modeled on the first 50 amino acids in the N-terminal portion of ANXA1 (CR-AnxA1_2–50_), and that binds specifically to FPR2/ALX, and exerts key pro-resolving actions in different inflammatory conditions (12–14).

Cardiopulmonary bypass (CPB) with deep hypothermic circulatory arrest (DHCA) is routinely performed during cardiac surgery for repair of thoracic aortic disease or complex congenital cardiac defects. Although this procedure remains necessary to maintain circulation of blood and oxygen while repairing the heart, it contributes to profound perturbations in inflammatory, hemostatic, and oxidative stress pathways, collectively implicated in the pathogenesis of perioperative cerebral injury (15–17). This inflammatory response is specifically activated *via* several pathways: the contact activation by the foreign surface of the CPB circuit, surgical trauma as well as the effect of ischemia–reperfusion (I/R) injury, and endotoxemia (18). Further, its effects have been related to central nervous system injury, including complications like delirium and postoperative cognitive dysfunction (19). Systemic inflammation after both cardiac and non-cardiac surgery can affect the brain *via* neuroinflammatory processes that are amplified by circulating pro-inflammatory cytokines in blood and cerebrospinal fluid (20–23) and localized neuronal impairments (24, 25).

We previously discovered a novel ANXA1 peptidomimetic (ANXA1sp or Ac-QAW) that suppresses human colon cancer growth *via* modulation of NF-κB activation (26). In the current study, we tested the effects of ANXA1sp on postoperative neuroinflammation and cognitive changes in an established rat model of CPB with DHCA and hypothesized that its pro-resolving mechanisms following I/R injury are mediated *via* attenuation of microglial activation.

## Materials and Methods

### Animals

The experimental protocol was approved by the Duke University Animal Care and Use Committee. All procedures were in accordance with the guidelines of the National Institutes of Health for animal care (Guide for the Care and Use of Laboratory Animals, Health and Human Services, National Institutes of Health Publication No. 86-23, revised 1996). Studies were conducted on adult male Sprague-Dawley rats (age 14–16 weeks; weight 400–450 g; Charles River Laboratories, Wilmington, MA, USA). Rats were housed (two animals per cage) in a 12-h light/dark cycle environment with free access to food and water. Rats were acclimated for at least 1 week before starting any experiment.

### Drug Treatments

Annexin A1 biomimetic tripeptide (ANXA1sp or Ac-QAW, Ac = acetyl, MW = 445.47 Da) was synthesized and purified (>98% purity) by GenScript (Piscataway, NJ, USA). The peptide was suspended in 100% DMSO. For *in vivo* experiments, this stock solution was diluted in saline to a final dose of 1 mg/kg ANXA1sp and a concentration of 1% DMSO. For *in vitro* experiments, the ANXA1sp–DMSO stock solution was diluted with culture medium to final concentrations ranging from 5 to 100 µM ANXA1sp. Vehicle control was 1% DMSO in saline for *in vivo* studies, and 1% DMSO in culture medium for *in vitro* studies. ANXA1sp treatment solutions were prepared fresh immediately before use for *in vivo* and *in vitro* experiments.

#### Short-term Survival Groups (3, 6, and 24 h)

Rats were randomly assigned to six groups (*n* = 5/group) and terminated for histologic and biochemical analyses at 3, 6, or 24 h after CPB/DHCA. Rats received ANXA1sp (1 mg/kg iv) or vehicle (1% DMSO iv) in 1 mL saline 1 h before CPB and 1 h after reperfusion. Rats in the 24-h survival group were also treated at 6 h after reperfusion. All treatments were administered in a blinded manner.

#### Long-term Survival Group (Day 7 Post Operation)

Rats were randomly assigned to two groups (*n* = 10/group) and treated as described above and then daily (ip) up to day 7 post operation. After neurologic and cognitive assessments on day 7 post operation, animals were terminated.

### Cardiac Surgery with CPB/DHCA

Fasted rats were anesthetized with isoflurane, intubated, and cannulated for CPB and DHCA without sternotomy, to allow for long-term survival, as previously described (27). Routine physiologic parameters, and pericranial and rectal temperature were continuously monitored. The heparinized CPB circuit consisted of a venous reservoir, a peristaltic pump, a custom-designed membrane oxygenator, and a flow probe. Lung ventilation was stopped for the entire period of CPB/DHCA. Following heparin administration, CPB was initiated at a flow rate of 160–180 mL/kg/min, which was then decreased as the animals were cooled over 30 min to a target pericranial temperature of 18°C. After reaching 18°C, the rats were subjected to DHCA, which was confirmed by electrocardiographic asystole and absence of any measurable MAP. After 60 min of DHCA, CPB was reinstituted, and rats were rewarmed over 30 min to a pericranial temperature of 34°C. CPB was then terminated, and mechanical ventilation resumed. After 2 h of continuous monitoring, rats were extubated, and recovered in a warmed oxygen-enriched environment with free access to water. Rats in the sham group (*n* = 3/group) were cannulated without exposure to CPB/DHCA; naïve rats were sacrificed under 5% isoflurane.

To harvest the brain, rats were re-anesthetized, intubated, and mechanically ventilated. One sample of brain tissue was immediately fixed in 10% buffered formalin and paraffin-embedded for immunostaining. The remaining brain tissue was frozen in liquid nitrogen and stored at −80°C until further use. Blood samples from each animal were also collected and stored at −80°C until analysis.

### Immunostaining of Microglia

Staining was performed on slices (20 µm thick) of the paraffin-embedded brain tissue samples using ionizing calcium-binding adaptor molecule 1 (Iba1) rabbit antibody (Wako Chemicals USA Inc., Richmond, VA, USA). For antigen retrieval, tissue slices were incubated with 10 mM citrate buffer, pH 6.0, for 5 min at 100°C. After the buffer solution cooled to room temperature (RT), slices were washed, and then blocked with 10% normal goat serum for 60 min at RT. Slices were then incubated with primary rabbit anti-Iba1 primary antibody (1:200) overnight at 4°C. After three washes with PBS, the slices were incubated with goat anti-rabbit secondary antibody conjugated with Alexa Fluor 488 (1:500, Invitrogen, Carlsbad, CA, USA) for 60 min at RT. Images were captured on a fluorescence microscope (Leica DM IRB, Germany) using a 10×/0.3 PH objective at 1.5-fold magnification. For quantification the total number of Iba1-positive cells was determined in five representative areas of the cerebral cortex (retrosplenial and posterior parietal cortex) and the hippocampus (CA1–CA3 area). Automated imaging and high-content analysis of microglia were done on the Cellomics ArrayScan IV platform and instrument (Thermo Fisher Scientific) using the Target Activation algorithm module optimized for object size, object shape, and fluorescence intensity to identify Iba1 positive cells by soma size (28). Microglial morphology was evaluated using a 4-scale classification method based on (29). Cells were classified based on their overall morphology as (1) round/amoeboid microglia, (2) stout microglia, (3) thicker longer ramifications, and (4) thinner ramifications by an investigator blinded to the experimental groups.

### Cell Death Assessment

Apoptosis was determined by terminal deoxynucleotidyl nick-end labeling (TUNEL) per assay manufacturer’s protocol (Roche Diagnostics, Indianapolis, IN, USA). Briefly, sections of the paraffin-embedded brain tissue sample (5 µm thick) were deparaffinized using xylene and descending grades of ethanol, and pretreated with microwave radiation (350 W, in 200 mL of 0.1 M Citrate buffer, pH 6.0) for 5 min. Tissue sections were then incubated with terminal deoxynucleotidyl transferase (TdT) for 1.5 h at 37°C and then rinsed with PBS. Slides of five representative areas of the retrosplenial and posterior parietal cortex and CA1 area of the hippocampus were mounted using UltraCruz™Mounting Medium with DAPI (Santa Cruz Biotechnology, Santa Cruz, CA, USA). Negative controls were incubated in label solution without TdT. A separate set of sections was stained with acid fuchsin–celestine blue to identify possible necrotic cells. Cell counting was performed in a blinded manner across five representative areas of the cerebral cortex and CA1 areas using a fluorescence microscopy (Leica DM IRB, Germany) with a 20×/0.4 PH objective at 1.5-fold magnification. Data obtained in every field were added together to make a final data count for each slide and expressed as percentage of total cell number within the relevant fields. For *in vitro* cell death assessment, cell culture medium (for necrosis) and cell lysate (for apoptosis) were assayed using the Cell Death Detection ELISA^PLUS^ per manufacturer’s protocol to measure cytoplasmic histone-associated DND fragments (momo- and oligonucleosomes) as previously described (30).

### Western Blots

Frozen brain samples were homogenized and protein quantified by BCA assay (Thermo Fisher Scientific). Western blotting was performed using SDS-PAGE 4–15% gradient gels (Bio-Rad) with the following antibodies: rabbit polyclonal against phosphor-p65; and ANXA1 (all from Cell Signaling Technology, Danvers, MA, USA). The bands were detected by Super-Signal West Dura Extended Duration Substrate (Thermo Scientific, Rockford, IL, USA). Band intensities of phosphor-p65 or ANXA1 were normalized with a loading control of β-actin.

### Neurologic Evaluation

On day 3 and day 7 post operation, rats underwent standardized functional neurologic testing by an observer blinded to group assignment, using an established neurologic scoring system that evaluates motor deficit (31). Briefly, rats were first placed on a 35 cm × 31.25 cm screen (grid size 0.6 cm × 0.6 cm) that could be rotated from horizontal (0°) to vertical (90°). The length of time that the rat could hold onto the screen after being rotated from 0 to 90° was recorded to a maximum of 15 s (0–3). Rats were then tested for balance on a horizontal wooden rod, and the time lapse before falling off the rod was recorded to a maximum of 30 s (0–3). Finally, rats underwent a prehensile traction test, and the length of time that the rat could cling to a horizontal rope was recorded to a maximum of 5 s (0–3). Animals received a score for each of the three tests. The final score was the sum of the individual test scores, with 0 the best score, and 9 the worst score.

### Morris Water Maze

The Morris water maze consisted of a pool of water (27°C), 1.5 m in diameter and 30 cm deep, with a hidden platform submerged 3 cm below the surface in one quadrant, and a computerized video tracking system (EthoVision^®^; Noldus, Wageningen, The Netherlands) (32). Rats were placed in the water in a dimly lit room with visual clues around the maze. The time to locate the submerged platform (defined as the escape latency) was measured. From day 3 through day 7 post operation, rats underwent daily testing in the water maze. Four trials were performed each day with an intertrial interval of 10 min. Each trial started in a different quadrant and was limited to 90 s of water exposure. A probe trial was performed on the last day of testing, and the submerged escape platform was removed from the water maze.

### BV2 Cell Culture and Hypoxic Exposure

Immortalized murine microglial cell line BV2 were maintained in Dulbecco’s modified Eagle’s medium (DMEM high glucose) containing 10% fetal bovine serum, 1% penicillin, and 1% streptomycin in a 37°C humidified incubator with 5% CO_2_ (balanced with air). Confluent cultures were passaged by trypsinization. After incubating for 24 h, cells were exposed to ANXA1sp (0, 5, 10, 20, 30, 40, 50, or 100 µM) for 1 h. Cells were then subjected to 2 h oxygen–glucose deprivation (OGD: DMEM no glucose, 85% N_2_/10% H_2_/5% CO_2_) in an OGD chamber (Farma Scientific), followed by reoxygenation for 3, 6, or 24 h in a 37°C growth incubator with 5% CO_2_ (balanced with air). Cells treated with 1% DMSO in the medium served as vehicle control. At the end of each time point, cells and culture supernatant were harvested for further analysis.

### Cell Viability/MTT Assay

Cell viability was determined by MTT (3-[4,5-dimethylthiazol-2-yl]-2,5 diphenyltetra-zolium bromide) assay per manufacturer’s protocol (Sigma-Aldrich, St. Louis, MO, USA). Briefly, a volume of MTT stock solution (5 mg/mL) equal to one-tenth the original culture volume was added to each culture to be assayed. After incubating for 3 h, cells were centrifuged at 800 *g* for 5 min, and the medium was removed. The formazan crystals were dissolved/solubilized in acidic isopropanol (0.04–0.1 N HCl in absolute isopropanol). Absorbance of converted dye was measured at a wavelength of 570 nm with background subtraction at 630–690 nm. Results were presented as cell viability (%) = average O.D. of treatment wells/average O.D. of vehicle-control wells.

### NF-κB DNA Binding Activity

Nuclear proteins were extracted from BV2 microglia or cerebral tissues per manufacturer’s protocol (Nuclear Extraction Kit, Panomics, Santa Clara, CA, USA). Protein concentration of nuclear extracts was measured using the BCA assay (Thermo Fisher Scientific, Grand Island, NY, USA). NF-κB DNA binding activity was assessed using a quantitative detection kit (Transcription Factor Assay Kit, Cayman Chemical, Ann Arbor, MI, USA). According to the manufacturer’s protocol, the 96-well plates were pre-coated with the specific double-stranded DNA sequence that contains the transcription factor NF-κB (p65) response element. Approximately 10 µg nuclear protein was incubated in the coated plate at RT for 1 h while rocking the plate gently at 150 rpm. After washing, NF-κB (p65)-specific primary antibody (1:100 dilution) was added, followed by horseradish peroxidase-labeled secondary antibody (1:100 dilution). The absorbance was read at 450 nm on a microplate reader.

### Cytokine Measurement

The concentrations of TNF-α and IL-6 in cell media, plasma, and brain homogenates were measured using rat-specific ELISA kits per manufacturer’s protocol (Thermo Fisher Scientific, Grand Island, NY, USA). The plasma was obtained by centrifugation at 2,000 *g* for 10 min at 4°C, and stored at −80°C until use. Brain homogenates were separated by centrifugation at 14,000 *g* for 10 min at 4°C to remove cellular debris. Change in absorbance in every well was detected at 450 nm on a microplate reader. All measurements were performed in triplicate.

### Myeloperoxidase (MPO) Measurement

Myeloperoxidase activity in brain tissue, whole cell lysates, and plasma was assessed using ELISA with a rat-specific MPO assay kit per manufacturer’s protocol (HK105, HyCult Biotechnology, Uden, The Netherlands).

### Confocal Microscopy

After deparaffinization, sections of the brain tissue sample were treated with 10 mM citrate buffer (pH 6.0) for antigen retrieval. After blocking with 10% normal goat serum at RT for 1 h, the sections were incubated with rabbit anti-ANXA1 antibody (1:500) and mouse anti-NF-κB p65 (1:500, Santa Cruz Biotechnology, Santa Cruz, CA, USA) at 4°C overnight. The sections were then incubated with Alexa Fluor 488-conjugated goat anti-rabbit IgG (1:500; Invitrogen, Carlsbad, CA, USA) and Alexa Fluor 550-conjugated goat anti-mouse IgG (1:500; Invitrogen, Carlsbad, CA, USA) at RT for 1 h. After washing with PBS, slides of the sections were prepared and mounted using UltraCruz™ Mounting Medium with DAPI (Santa Cruz Biotechnology, Santa Cruz, CA, USA) to detect nuclei.

For *in vitro* confocal microscopy, adherent BV2 cells grown on coverslips were fixed by adding 4% paraformaldehyde to the medium, and incubating for 15 min at RT. After rinsing with PBS, coverslips were permeabilized in freshly prepared 0.1% Triton X-100 and 0.1% sodium citrate for 10 min at RT. After washing and blocking with 10% normal goat serum and 1% BSA for 1 h at RT, coverslips were incubated with rabbit anti-ANXA1 antibody (1:1,000, Santa Cruz Biotechnology, Santa Cruz, CA, USA) and mouse anti-NF-κB p65 (1:500, Santa Cruz Biotechnology, Santa Cruz, CA, USA) at 4°C overnight. Coverslips were then incubated with Alexa Fluor 488-conjugated goat anti-rabbit IgG (1:1,000; Invitrogen, Carlsbad, CA, USA) and Alexa Fluor 555-conjugated goat anti-mouse IgG (1:1,000; Invitrogen, Carlsbad, CA, USA) at RT. Coverslips were mounted using UltraCruz™ Mounting Medium with DAPI (Santa Cruz Biotechnology, Santa Cruz, CA, USA) to detect nuclei. Images were captured on a Leica SP5 confocal microscope (Leica Microsystems, Germany) using a 63×/1.25-0.75 Plan APO oil objective, and the images were analyzed by NIH ImageJ software (version 1.51).

### Statistical Analysis

Statistical analysis was performed using Statview Software (version 5, SAS Institute, Cary, NC, USA) and graphs presented with Prism 7 (GraphPad Software, San Diego, CA, USA). Results were expressed as mean ± SD. Morris water maze performance was compared by repeated measures analysis of variance, with time as the repeated measure and Fisher’s least significance difference *post hoc* test. The Mann–Whitney *U* test was used to compare neurologic scores between groups at each recovery interval. Parametric values, including physiologic values, data from ELISA, western blots, as well as numbers of apoptotic and necrotic cells, were compared between groups using the Student’s *t*-test. One-way or two-way analysis of variance followed by Tukey’s or Sidak’s multiple comparison *post hoc* was used as defined in the figure legends. Statistical significance was assumed when *P* < 0.05.

## Results

No deaths were reported after CPB/DHCA in this study. Intraoperative physiologic values (MAP, Hct, glucose, pH, PaCO_2_, PaO_2_, ${\text{HCO}}_{3}^{-}$, and pericranial temperature) in rats treated with vehicle or ANXA1sp are summarized in Table 1. Intergroup comparisons show no statistical differences, with all values within normal limits.

**Table 1:** Intraoperative physiologic data.

Parameter	Group	Pre-CPB	30 min CPB	60 min DHCA	10 min Reperfusion	End of CPB	120 min P-CPB
MAP (mmHg)	Vehicle	70 (7)	30 (4)	–	72 (25)	88 (11)	88 (6)
	ANXA1sp	66 (7)	29 (4)	–	49 (12)	78 (22)	81 (17)
Temp. (°C)	Vehicle	34.1 (0.8)	19.3 (0.6)	15.0 (0.1)	21.9 (3.0)	33.2 (0.6)	36.0 (0.8)
	ANXA1sp	34.2 (0.3)	19.1 (0.5)	15.0 (0.1)	22.0 (2.3)	33.8 (0.5)	36.4 (0.3)
Glucose (mg/dL)	Vehicle	124 (40)		–		190 (44)	143 (42)
	ANXA1sp	123 (46)		–		170 (36)	116 (38)
PH	Vehicle	7.36 (0.08)	7.46 (0.05)	–	7.57 (0.08)	7.28 (0.05)	7.36 (0.03)
	ANXA1sp	7.38 (0.02)	7.48 (0.09)	–	7.51 (0.15)	7.29 (0.02)	7.40 (0.05)
PO_2_ (mmHg)	Vehicle	227 (46)	516 (47)	–	374 (69)	245 (114)	256 (71)
	ANXA1sp	253 (93)	474 (77)	–	257 (211)	170 (138)	247 (126)
PCO_2_ (mmHg)	Vehicle	53.2 (13.1)	40.2 (3.8)	–	28.6 (4.6)	55.8 (8.7)	52.0 (4.4)
	ANXA1sp	49.4 (5.1)	38.6 (9.7)	–	31.2 (12.0)	53.0 (5.1)	48.6 (6.1)
${\text{HCO}}_{3}^{-}$ (meq/L)	Vehicle	29.4 (1.9)	24.6 (0.8)	–	25.7 (2.3)	25.7 (2.6)	29.0 (1.0)
	ANXA1sp	29.2 (1.6)	28.3 (1.2)	–	23.5 (1.7)	25.2 (1.9)	29.8 (1.4)
Hct (%)	Vehicle	41.4 (2.3)	25.0 (0.7)	–	25.0 (1.2)	28.0 (0.8)	39.0 (2.9)
	ANXA1sp	41.6 (1.9)	24.2 (0.8)	–	25.2 (0.8)	27.4 (2.6)	38.4 (2.1)

*Values in brackets represent SD (– indicates no values due to the bypass machine being on)*.

*CPB, cardiopulmonary bypass; DHCA, deep hypothermic circulatory arrest*.

### ANXA1sp Treatment Attenuates Neuroinflammation and Systemic Inflammation after CPB/DHCA

**Figure 1:**
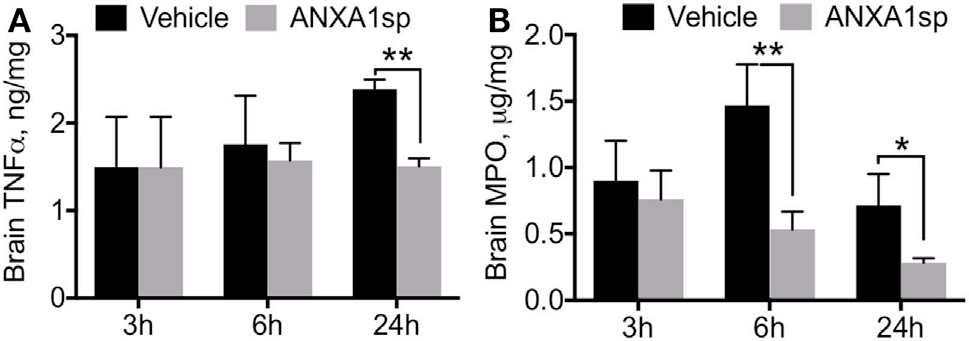
Cytokine levels in brain homogenates after cardiopulmonary bypass/deep hypothermic circulatory arrest by treatment group. ELISA assays revealed time-dependent reductions in brain levels of TNFα **(A)** and myeloperoxidase **(B)** in ANXA1sp-treated rats, with significant effects at 6 and 24 h post reperfusion. Data are presented as mean ± SD (*n* = 3–5 rats/group). *P < 0.05, **P < 0.01 compared to vehicle controls, analyzed with unpaired *t* test.

**Figure 2:**
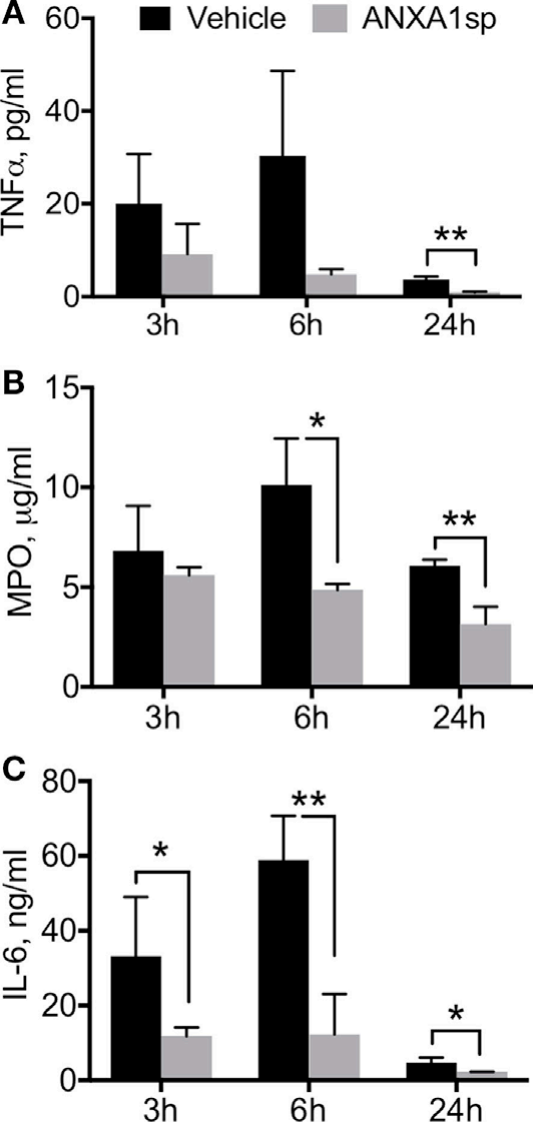
Circulating cytokine levels after cardiopulmonary bypass/deep hypothermic circulatory arrest by treatment group. ELISA assays of plasma levels of TNFα **(A)**, myeloperoxidase (MPO) **(B)**, and IL-6 **(C)** showed that time-dependent effects were noted in TNFα and MPO levels, with significant reduction in ANXA1sp rats by 24 h post reperfusion. IL-6 levels were significantly reduced in ANXA1sp-treated rats as early as 3 h post reperfusion. Data are presented as mean ± SD (*n* = 3–5 rats/group). *P < 0.05, **P < 0.01 compared to vehicle controls, analyzed with unpaired *t* test.

Neuroinflammation is a critical hallmark in several neurocognitive disorders (33). After CPB/DHCA, we found a significant increase in brain levels of key pro-inflammatory cytokines such as TNF-α and MPO (Figure 1). TNF-α was elevated 24 h after surgery, with protein levels significantly reduced following ANXA1sp treatment (Figure 1A, 12.39 ± 0.11 vs 1.50 ± 0.09, *P* < 0.01). Similarly, MPO levels were lower in ANXA1sp-treated rats at 6 and 24 h compared to controls (Figure 1B, 1.47 ± 0.31 vs 0.53 ± 0.14 at 6 h; 0.71 ± 0.24 vs 0.53 ± 0.14 at 24 h, *P* < 0.01). Given the known effects of CPB surgery on the systemic inflammatory response we also measured plasma levels of these pro-inflammatory cytokines and IL-6. Levels of TNF-α, MPO, and IL-6 were elevated after injury, peaking at 6 h and returning toward baseline at 24 h. ANXA1sp-treated rats had significantly blunted systemic inflammation (Figure 2). Although statistically significant changes were measured only at 24 h, both 3 and 6 h treated groups had lower levels of plasma TNF-α (Figure 2A, 3.63 ± 0.68 vs 0.88 ± 0.17 at 24 h, *P* < 0.01). Systemic levels of MPO were reduced at 6 and 24 h after treatment (Figure 2B, 10.12 ± 2.33 vs 4.87 ± 0.30, *P* < 0.05; 6.07 ± 0.31 vs 3.15 ± 0.86, *P* < 0.01), whereas IL-6 was reduced at all time points (Figure 2C, 33.20 ± 15.81 vs 11.83 ± 2.33 at 3 h, *P* < 0.05; 58.94 ± 11.79 vs 12.29 ± 10.80 at 6 h, *P* < 0.01; 4.71 ± 1.36 vs 2.27 ± 0.04 at 24 h, *P* < 0.05).

### Regulation of Microglial Activation and Cell Death by ANXA1sp after CPB/DHCA

**Figure 3:**
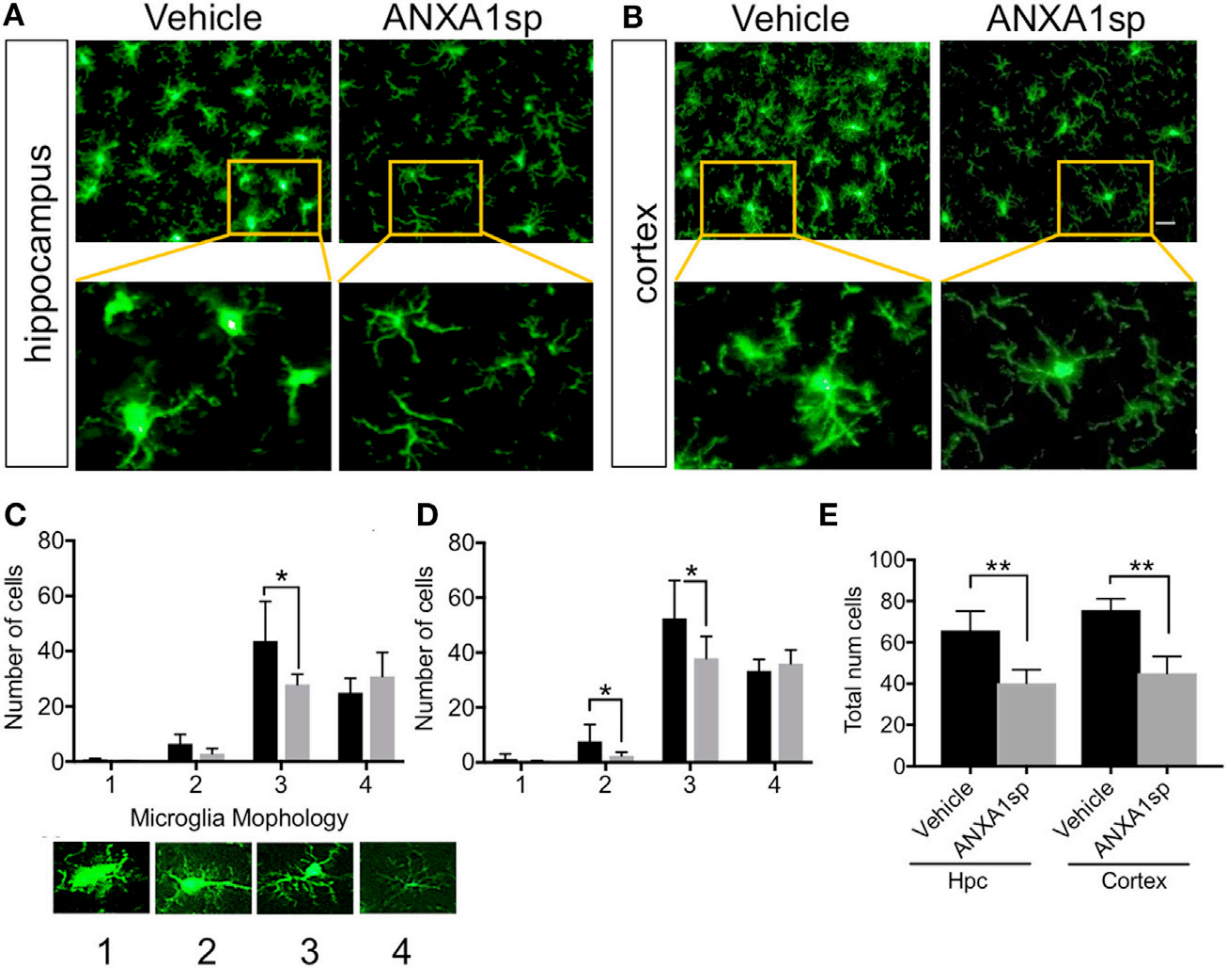
Microglial activation after cardiopulmonary bypass/deep hypothermic circulatory arrest and ANXA1sp or vehicle treatment. ANXA1sp significantly improved microglial morphology both in the hippocampus **(A)** and cerebral cortex **(B)** 24 h after CPB/DHCA. **(C,D)** Microglial morphology was quantified based on four morphological subtypes: 1. round/amoeboid microglia; 2. stout microglia; 3. microglia with thick long processes; and 4. microglia with thin ramified processes. **(E)** Overall microglial numbers in the hippocampus and cerebral cortex were reduced after surgery in ANXA1sp-treated rats. Scale bar: 20 µm. Data are presented as mean ± SD (*n* = 3–5 slides/tissue section from five rats per group). *P < 0.05 compared to vehicle controls, analyzed with two-way ANOVA Sidak’s multiple comparisons test **(C,D)** and **P < 0.01 with unpaired *t* test **(E)**.

Microglia are resident immune cells in the CNS that have key functions in homeostasis and disease development (34). Changes in microglial morphology are often associated with pathological states. Here we found that CPB/DHCA induced distinct changes in microglial activation both in the hippocampus CA1–CA3 area and retrosplenial and posterior parietal cortex (Figure 3). Using a method identified to characterize microglia morphology (29) we found ANXA1sp treatment (1 mg/kg) significantly attenuated microglial activation (Figures 3A,B). This was evidenced by reduced numbers of Iba1-positive cells in the hippocampus CA1–CA3 area (*P* < 0.01) and retrosplenial and posterior parietal cortex (*P* < 0.01) at 24 h post reperfusion (Figure 3A). This was particular significant for microglial with thicker processes (scale 3, Figure 3C) as well as stout microglia in the cortex (scale 2, Figure 3D). Overall, CPB/DHCA surgery did not induce significant round/ameboidal microglia (scale 1) at this time point. However, overall number of microglia both in the hippocampus and cerebral cortex were reduced using automated imaging and high-content analysis of soma size (Figure 3E).

**Figure 4:**
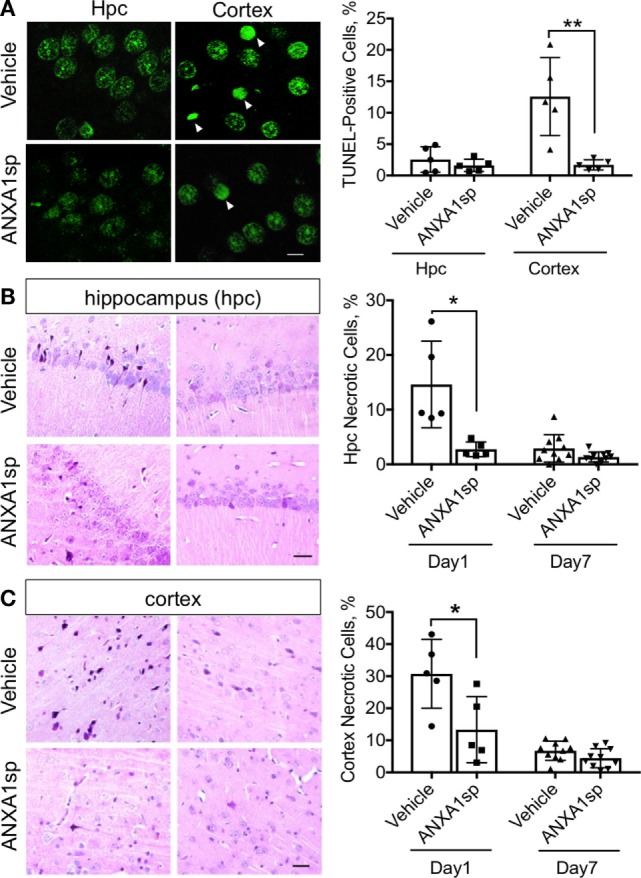
Apoptosis and necrosis in the CNS after cardiopulmonary bypass/deep hypothermic circulatory arrest and ANXA1sp or vehicle treatment. **(A)** Terminal deoxynucleotidyl nick-end labeling staining and quantification of apoptotic bodies. Apoptosis was significantly reduced in the cerebral cortex of ANXA1sp-treated rats on day 1 post operation. **(B,C)** Necrosis was detected by acid fuchsin–celestine. Necrosis was reduced in a time-dependent fashion in the hippocampus and cerebral cortex of ANXA1sp rats, with levels returning to baseline by day 7 post operation. Arrowheads in panel A identify pyknotic positive cells. Scale bar: 20 µm. Data are presented as mean ± SD (*n* = 5–10 slides/tissue section from five rats per group). *P < 0.05, **P < 0.01 compared to vehicle controls, analyzed with unpaired *t* test.

Finally, ANXA1sp treatment was also associated with a significant reduction in TUNEL-positive cells in the cerebral cortex, but not in the hippocampus, at 24 h after CPB/DHCA (Figure 4A). Staining with acid fuchsin–celestine blue also revealed acidophilic neurons and possible necrosis in the hippocampus (Figure 4B) and cerebral cortex (Figure 4C) that was reduced in rats treated with ANXA1sp at day 1 and 7 post operation.

### *In Vivo* and *In Vitro* Modulation of NF-κB Activity by ANXA1sp

**Figure 5:**
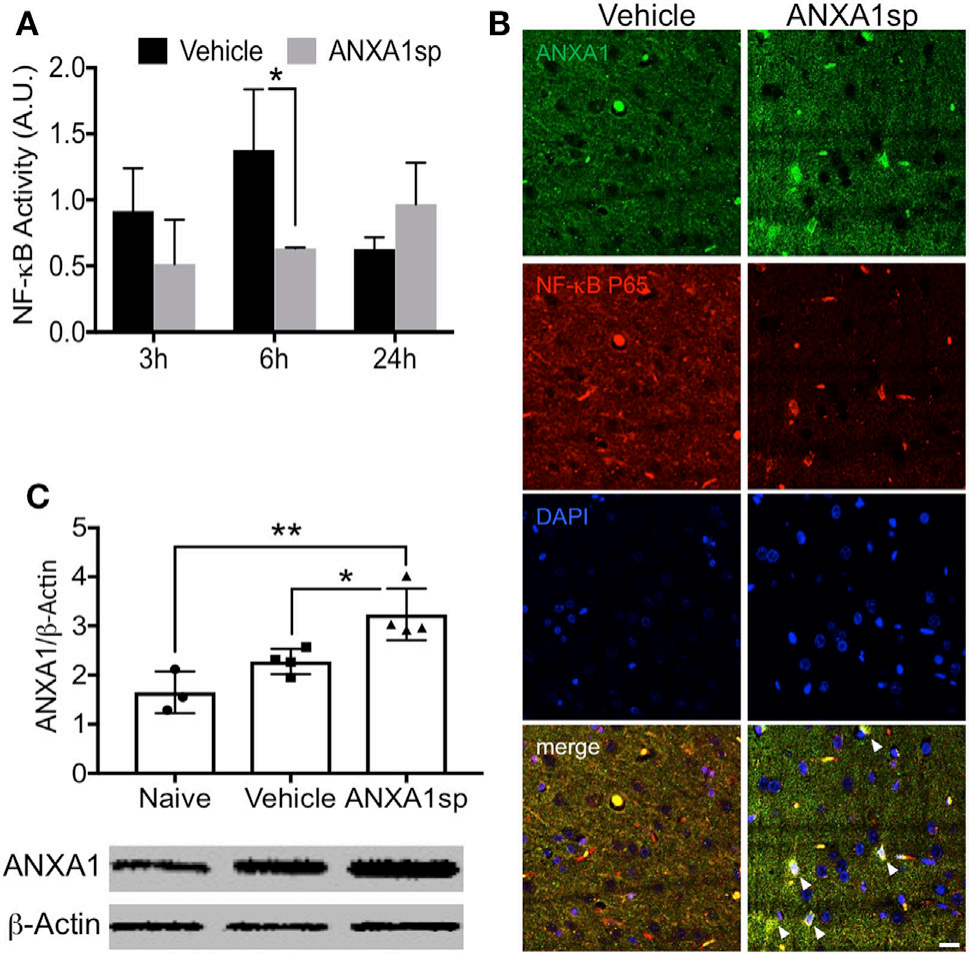
Modulation of cerebral NF-κB DNA binding activity and expression of ANXA1 after cardiopulmonary bypass/deep hypothermic circulatory arrest. **(A)** NF-κB DNA binding activity was significantly reduced in brain homogenates from ANXA1sp-treated rats at 6 h post reperfusion. **(B)** Co-localization of ANXA1 and NF-κB p65 (arrowheads) was visualized by double immunofluorescence staining and confocal microscopy. **(C)** ANXA1sp promoted expression of cerebral ANXA1 in rats at 24 h following CPB/DHCA. Scale bar: 20 µm. Data are presented as mean ± SD (*n* = 3–5 rats/group). *P < 0.05, **P < 0.01 compared to vehicle controls, analyzed with unpaired *t* test **(A)** or one-way ANOVA with Tukey’s multiple comparisons test **(C)**.

We previously demonstrated that both non-steroidal and steroidal anti-inflammatory drugs such as glucocorticoids and nitric oxide–aspirin, induce expression of ANXA1, which directly binds to the NF-κB p65 subunit, and thereby inhibits its activation in cancer models (26). Here, we found that NF-κB activity in the brain was significantly attenuated in the ANXA1sp-treated rats at 6 h after CPB/DHCA (Figure 5A). Notably, levels of NF-κB increased after CPB/DHCA (with vehicle) compared to sham and naïve rats (Figure S1 in Supplementary Material), hence we focused this study on the comparison between vehicle-treated and ANXA1sp-treated rats. Confocal microscopy in the cerebral cortex revealed ANXA1 co-localized with nuclear NF-κB p65 after treatment, suggesting a possible similar mechanism as earlier described in cancer models (26) (Figure 5B). Thus, we assessed protein levels of ANXA1 by western blot and found a significant increase in the expression following peptide administration compared to naïve- and vehicle-treated rats (Figure 5C, *P* < 0.01 vs naïve and *P* < 0.05 vs vehicle, respectively).

Given the effects of ANXA1sp on microglial activation after CPB/DHCA we then used immortalized murine microglial cell line BV2. BV2 cells were pretreated with ANXA1sp for 1 h, and then exposed to 2 h OGD (hypoxia) followed by 24 h reoxygenation, to mimic I/R injury from CPB in the rat model. No changes were observed in ANXA1sp-treated cells under normoxic or sham conditions. However, in cells subjected to hypoxia, we discovered that cell survival (by MTT assessment) was maximal after pretreatment with 30 µM ANXA1sp (Figure S2 in Supplementary Material). Thus, we used this dosage for the remainder of the *in vitro* studies reported here.

**Figure 6:**
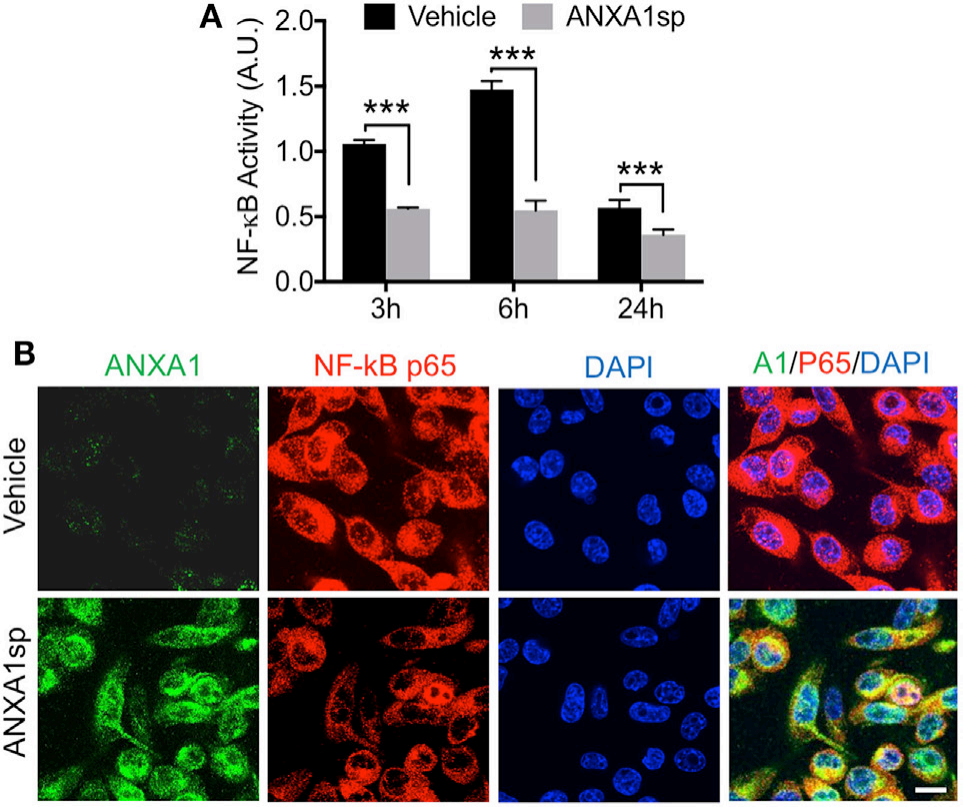
Modulation of NF-κB activation after oxygen–glucose deprivation in BV2 cells pretreated with 30 µM ANXA1sp or vehicle. **(A)** NF-κB activity based on the gel electrophoresis mobility shift assay was significantly reduced in a time-dependent fashion in ANXA1sp-treated cells. **(B)** After 24 h reoxygenation, co-localization of annexin A1 (ANXA1) and NF-κB p65 subunits was visualized by double immunofluorescence staining and confocal microscopy. Scale bar: 20 µm. Data are presented as mean ± SD (*n* = 3 independent experiments). ***P < 0.001 compared to vehicle controls, analyzed with unpaired *t* test.

**Figure 7:**
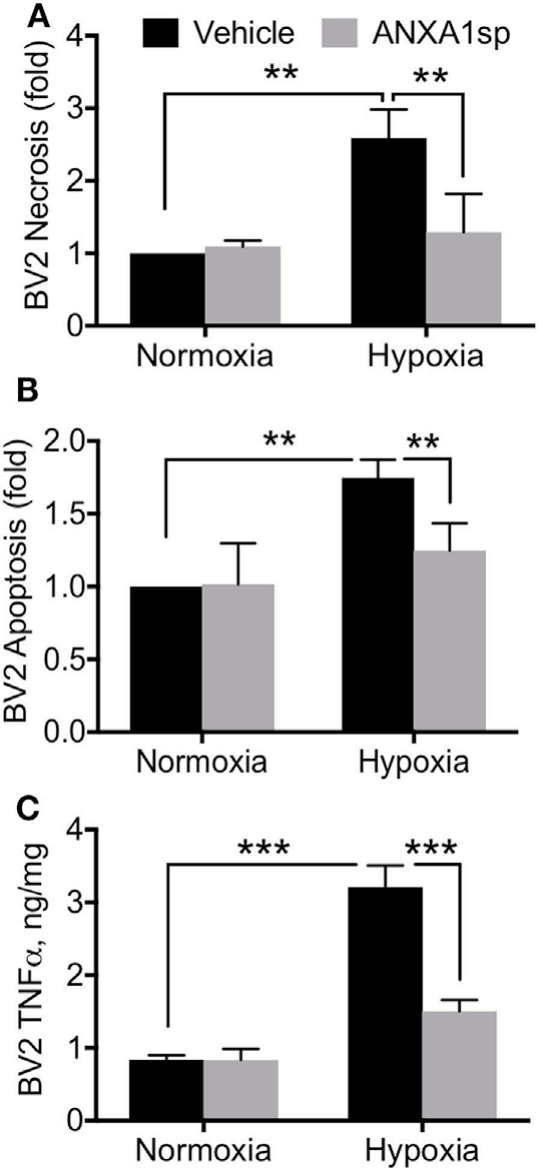
ANXA1sp reduced microglial cell death and TNFα. BV2 cells were pretreated with ANXA1sp or vehicle for 1 h, and then subjected to 2 h oxygen–glucose deprivation (OGD) followed by reoxygenation. Cell necrosis **(A)** and apoptosis **(B)** were assessed by ELISA assay. Following OGD, 30 µM ANXA1sp prevented both cell necrosis and apoptosis in ANXA1sp-treated BV2 cells. **(C)** TNFα levels in cell culture medium were also restored in cells pretreated with ANXA1sp. Data are presented as mean ± SD (*n* = 3 independent experiments). 30 µM ANXA1sp was selected as the optimal treatment based on the dose response (Figure S2 in Supplementary Material). **P < 0.01, ***P < 0.001 compared to vehicle controls, analyzed with unpaired *t* test or one-way ANOVA with Tukey’s multiple comparisons test.

Cells pretreated with ANXA1sp had lower levels of NF-κB DNA binding activity (based on the gel electrophoresis mobility shift assay) at all time points after OGD (Figure 6A). Using confocal microscopy we also observed increased levels of ANXA1 and co-localization with NF-κB p65 (Figure 6B). These findings corroborated our *in vivo* results. Further, both necrotic (Figure 7A, 2.58 ± 0.40 vs 1.29 ± 0.53, *P* < 0.01) and apoptotic (Figure 7B, 1.74 ± 0.12 vs 1.23 ± 0.18, *P* < 0.01) cell death following hypoxia reoxygenation were reduced after pretreatment with 30 µM ANXA1sp assessing oligosome formation as an index of DNA fragmentation by ELISA (30). TNFα release in culture media was also suppressed after pretreatment with ANXA1sp (Figure 7C, 3.21 ± 0.30 vs 1.51 ± 0.15, *P* < 0.001).

### Neurological and Neurocognitive Outcomes after CPB/DHCA and ANXA1sp Treatment

**Figure 8:**
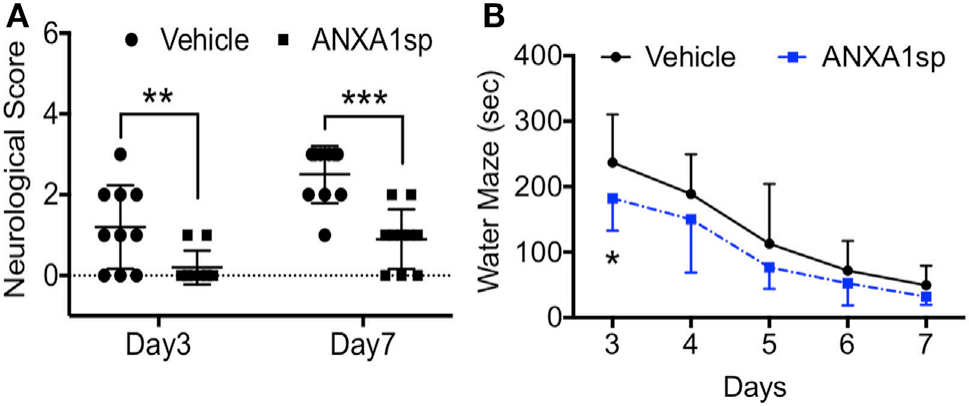
Neurocognitive outcomes after cardiopulmonary bypass/deep hypothermic circulatory arrest and ANXA1sp or vehicle treatment. **(A)** Neurologic scores were assessed on day 3 and day 7 post operation. Sensory-motor functions were significantly improved at both time points in ANXA1sp-treated rats. **(B)** Morris water maze testing was performed daily from day 3 through day 7 post operation. Learning abilities were significantly improved in ANXA1sp rats on day 3 post operation. Data are presented as mean ± SD (*n* = 10 rats/group). *P < 0.003, **P < 0.01, ***P < 0.001 compared to vehicle controls. Mann–Whitney *U* test was applied for the neurological score comparisons at each recovery interval. Repeated measures ANOVA with Fisher’s least significance difference *post hoc* test was used for Morris water maze analysis.

Finally, we evaluated neurobehavioral changes after CPB/DHCA and ANXA1sp treatment. Neurologic scores were assessed on day 3 and day 7 post operation and showed improved sensory-motor functions (including processing involving retrosplenial and posterior parietal cortex) in ANXA1sp-treated rats compared to vehicle-treated rats, with scores returning to baseline by day 7 post operation (Figure 8A). We used the Morris water maze to evaluate spatial learning and memory (involving hippocampal function). Fisher’s least significance difference *post hoc* test showed the cognitive function was significantly improved at day 3 of the water maze (Figure 8B, *P* < 0.003).

## Discussion

In this study, we evaluated the potential of a bioactive ANXA1 peptidomimetic to confer neuroprotection after cardiac surgery. Our findings show that systemic administration of ANXA1sp reduced brain and circulating levels of pro-inflammatory markers while improving neurocognitive outcomes following CPB/DHCA in rats. In particular, treated animals displayed significant decreases in microglial activation, NF-κB activation, and release of pro-inflammatory mediators in the CNS and systemically, as well as modulation of cell death in different brain regions after CPB/DHCA.

Annexin A1 was the first described member of the annexin superfamily, which includes 13 mammalian proteins with distinct biologic roles (35). It is widely expressed in different organs, and signals *via* the lipoxin A4 receptor (ALX). ANXA1 has profound effects on innate immunity including regulation of glucocorticoid activity by inhibiting eicosanoid synthesis and phospholipase A2 (PLA2) (36). The N-terminal domain of this molecule is pivotal in mediating several of its biologic functions as well as signaling *via* FPR receptors (37). These actions result in potent immunoregulatory effects, especially on inflammatory phagocytes and neutrophils by inhibiting their accumulation and migration. After splanchnic artery occlusion and reperfusion injury, treatment with ANXA1 N-terminal peptidomimetic (Ac_2–26_) reduced MPO activity and neutrophil infiltration into the reperfused tissue and thus, improved outcome after shock (38). Recently, using a model of middle cerebral artery occlusion and reperfusion in mice that recapitulates warm focal I/R injury, Vital et al. showed attenuation of cerebrovascular injury after administration of ANXA1 Ac_2–26_ (39). This study highlighted the importance of FPR2/ALX on neutrophils as a central player in controlling formation of neutrophil-platelet aggregates in the cerebral microcirculation post-I/R as a central mechanism for resolving neuroinflammation. Our findings complement those of Vidal et al. in a rat model of cardiac surgery-associated cold global cerebral I/R, with postoperative MPO levels significantly reduced in brain and plasma following ANXA1sp treatment (Figures 1B and 2B), suggesting a similar mode of action and attenuation of neutrophil infiltration into the CNS.

Changes in endothelial function have been described after cardiac and non-cardiac surgery (40–42), with translational relevance as well as significant implications related to the pathogenesis of postoperative delirium and cognitive dysfunction. Indeed, after CPB/DHCA, we previously reported changes in blood–brain barrier (BBB) permeability and tight junction protein expression in purified CNS capillaries (43). Although we cannot ascertain whether ANXA1sp exerts systemic and/or central effects, BBB opening after CPB provides direct access to the brain and several putative cellular targets. ANXA1sp is expressed by different cell types in the CNS, including neurons, microglia, and astrocytes (44). Moreover, ANXA is a critical regulator of BBB integrity by stabilizing tight junction expression and is often downregulated in disorders such as multiple sclerosis (45) and Alzheimer’s disease (46). Here, we found sound evidence for potent neuroprotective effects of ANXA1sp, including reduction in neuroinflammation (Figure 1), microglial activation (Figure 3), cell death (Figure 4), and overall improved neurobehavioral outcomes (Figure 8). Some of these effects may be systemically mediated with direct actions at the inflammatory site given the peripheral route of drug administration in this study. In fact, plasma levels of pro-inflammatory cytokines including TNF-α and IL-6 were reduced as early as 3 h after reperfusion, with levels returning to baseline by 24 h (Figure 2). This is consistent with the anti-inflammatory effects, as well as direct myocardial, protection of ANXA1 in other models of cardiac injury (47, 48).

Our current study focused on remote effects of CPB and DHCA on the CNS by evaluating a potential role for ANXA1sp in resolving neuroinflammation. We found that cell necrosis and apoptosis were reduced in different brain regions after CPB/DHCA in ANXA1sp-treated rats. In an earlier study on ANXA1, similar findings were reported after spinal cord injury through inhibition of caspase-3 and PLA2 activity (49). Further, ANXA1 in microglia facilitates clearance of apoptotic neurons after contact with a neurotoxin (50).

Microglia are central to the onset and progression of inflammation in the CNS. Although the function and exact role of these cells is highly dependent on activation state, reactive microglia contribute to neuroinflammation and a maladaptive response that contributes to neuronal dysfunction (33). After exposure to CPB and DHCA, we found changes in numbers and morphology of microglia in different brain areas, including the hippocampus (Figure 3). Microglial activation has been reported in orthopedic (51), vascular (52), abdominal (53), and cardiac (54) surgery-induced neurocognitive disorders. Although the mechanisms that contribute to microglial activation are multifactorial and include both humoral and neuronal signaling, modulation of ANXA1 is a promising target for intervention.

Annexin A1 is abundant in microglia (55), and we found that ANXA1sp boosts expression of ANXA1 in these cells possibly facilitating resolution of neuroinflammation (Figures 3 and 7). Our *in vivo* and *in vitro* data demonstrate a key role for NF-κB activation in microglial cells, and NF-κB activation represents a key regulatory gene for *de novo* synthesis of pro-inflammatory cytokines as well as cell death processes. Although, we cannot ascertain if microglia are the primary and sole target of ANXA1sp, this small peptidomimetic is likely to exert several effects both on peripheral and central inflammatory processes. Importantly, post-I/R NF-κB activation was dampened after treatment with ANXA1sp (Figures 5–7), and TNF-α and IL-6 levels were reduced accordingly in both brain tissue and plasma in a time-dependent fashion after CPB/DHCA (Figures 1 and 2). These findings suggest then, that modulation of NF-κB activation may reduce neuronal damage and improve behavioral outcomes after cardiac surgery.

Several protective effects of ANXA peptidomimetics have been described in different models [reviewed in Ref. (56)]. We previously reported anti-inflammatory effects of our ANXA1sp tripeptide on NF-κB inhibition in models of colon cancer (26). In this surgical model, ANXA1sp not only reduces NF-κB DNA binding activity, but also increases levels of ANXA1, which can bind directly to NF-κB p65 to further inhibit its transcriptional activity (Figure 5). These findings are relevant and possibly unique since the N-terminal domain sequence of this tripeptide (Ac-Gln^10^–Ala^11^–Trp^12^) has been shown to have greater binding affinity for FPR2/ALX (57). In general, FPR2/ALX shows high promiscuity in terms of ligand recognition, and thus possesses very complex functional properties including both promotion of resolution and pro-inflammatory effects (58). This may be important because even though other peptidomimetics, including nanoparticles encapsulating ANXA1 mimetic peptide Ac_2–26_ (59), SuperAnxA1 (60), and CR-AnxA1_2–28_ (14), regulate efferocytosis and neutrophil activity, ALX agonists can activate other specialized pro-resolving signaling and extend therapeutic effects (61). Currently, surgery-induced neurologic complications such as delirium and postoperative cognitive dysfunction have no effective therapy. Between 14% (62) and 50% (63) of cardiac surgery patients suffer from postoperative neurocognitive impairments, with effects lasting up to several years (64). Therapeutic strategies that reduce and promote resolution of neuroinflammation may limit these complications without significantly affecting homeostatic and reparative processes (65).

In the current study, ANXA1sp was systemically administered, and thus, we cannot verify the exact site of action of this small peptide. Although we observed significant anti-inflammatory changes both systemically and in the CNS, we cannot yet assign causal effects. For our *in vitro* experiments we used an immortalized murine microglial cell line (BV2) as a suitable alternative to primary microglia (50, 66), however, the role ovvf FPR2/ALX signaling in microglial activation or monocytes trafficking through the BBB were not formally evaluated in this study. Given the critical role of ANXA1 in regulating BBB integrity, it is possible that regulation of endothelial function after surgery protects against secondary effects in the CNS. We used a clinically relevant rat model of CPB and DHCA to study secondary effects on the CNS, yet to allow for long-term survival, median sternotomy, direct cardiac cannulation, and opening of cardiac cavities were not performed. Extended exposures to anesthesia, such as sevoflurane, without surgical manipulation downregulated ANXA1 expression in endothelial cells, which contributed to cognitive deficits (67). Here, we found that expression of ANXA1 was also increased following ANXA1sp treatment (Figure 5C), and although the signaling mechanisms *via* FPR2/ALX must be further refined. It remains unclear if treatment with this ANXA1 peptidomimetic boosts endogenous levels of ANXA1 or simply contributes to the existing pool of pro-resolving factors, thus facilitating the resolution cascade.

Taken together these findings demonstrate the potential for exploiting innate neuroprotective mechanisms to minimize cerebral I/R damage in general and that novel stable activators of this pathway may serve as resolution-targeting strategies to prevent or limit perioperative cerebral injury and associated neurocognitive complications.

## Ethics Statement

The experimental protocol was approved by the Duke University Animal Care and Use Committee. All procedures were in accordance with the guidelines of the National Institutes of Health for animal care (Guide for the Care and Use of Laboratory Animals, Health and Human Services, National Institutes of Health Publication No. 86-23, revised 1996).

## Author Contributions

ZZ, QM, and MP designed and performed research; BS, DL, GM, JM, MP, and NT contributed new reagents/analytic tools; ZZ, QM, MP, and NT analyzed data; ZZ, QM, and NT wrote the manuscript; ZZ and QM contributed equally to this study. All the authors read and approved the final manuscript.

## Conflict of Interest Statement

ZZ, NT, QM, MP, and JM are coinventors on patents filed through Duke University on the therapeutic use of ANXA1sp. All other authors declare no conflict of interest.

## Supplementary Material

Figure S1 Expression of NF-κB in naive, sham, and cardiopulmonary bypass with deep hypothermic circulatory arrest.

Figure S2 Cell viability measured by MTT assay at 24 h OGD/reoxygenation.
