# Supplementary figures and images for: Neuroprotective Effects of Annexin A1 Tripeptide after Deep Hypothermic Circulatory Arrest in Rats
Source: Front Immunol. 2017 Aug 30;8:1050. doi: 10.3389/fimmu.2017.01050 (PMC5582068; doi:10.3389/fimmu.2017.01050)

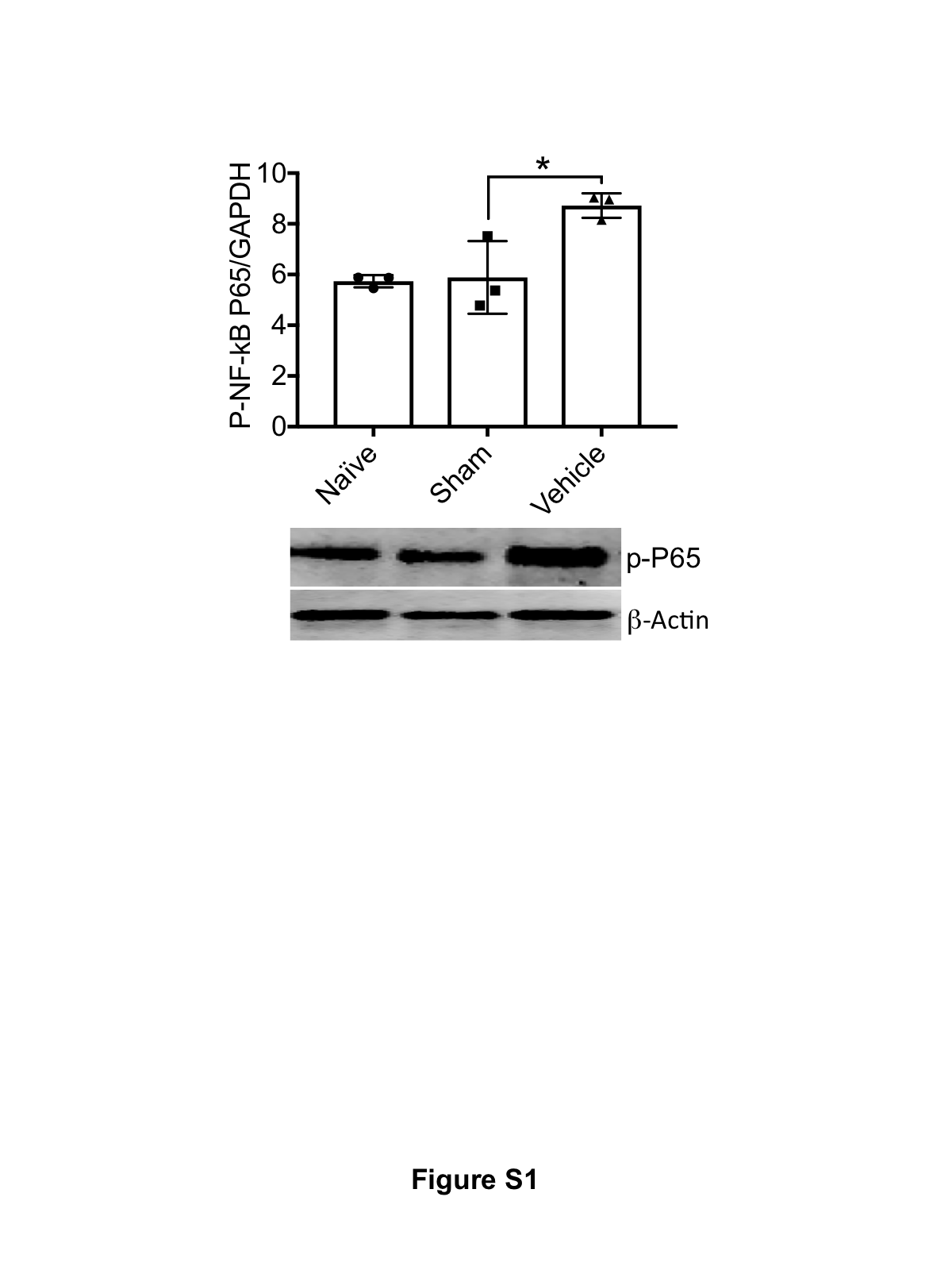

Supplement: Figure S1 — Expression of NF-κB in naive, sham, and cardiopulmonary bypass with deep hypothermic circulatory arrest. [file Image_1.TIFF]

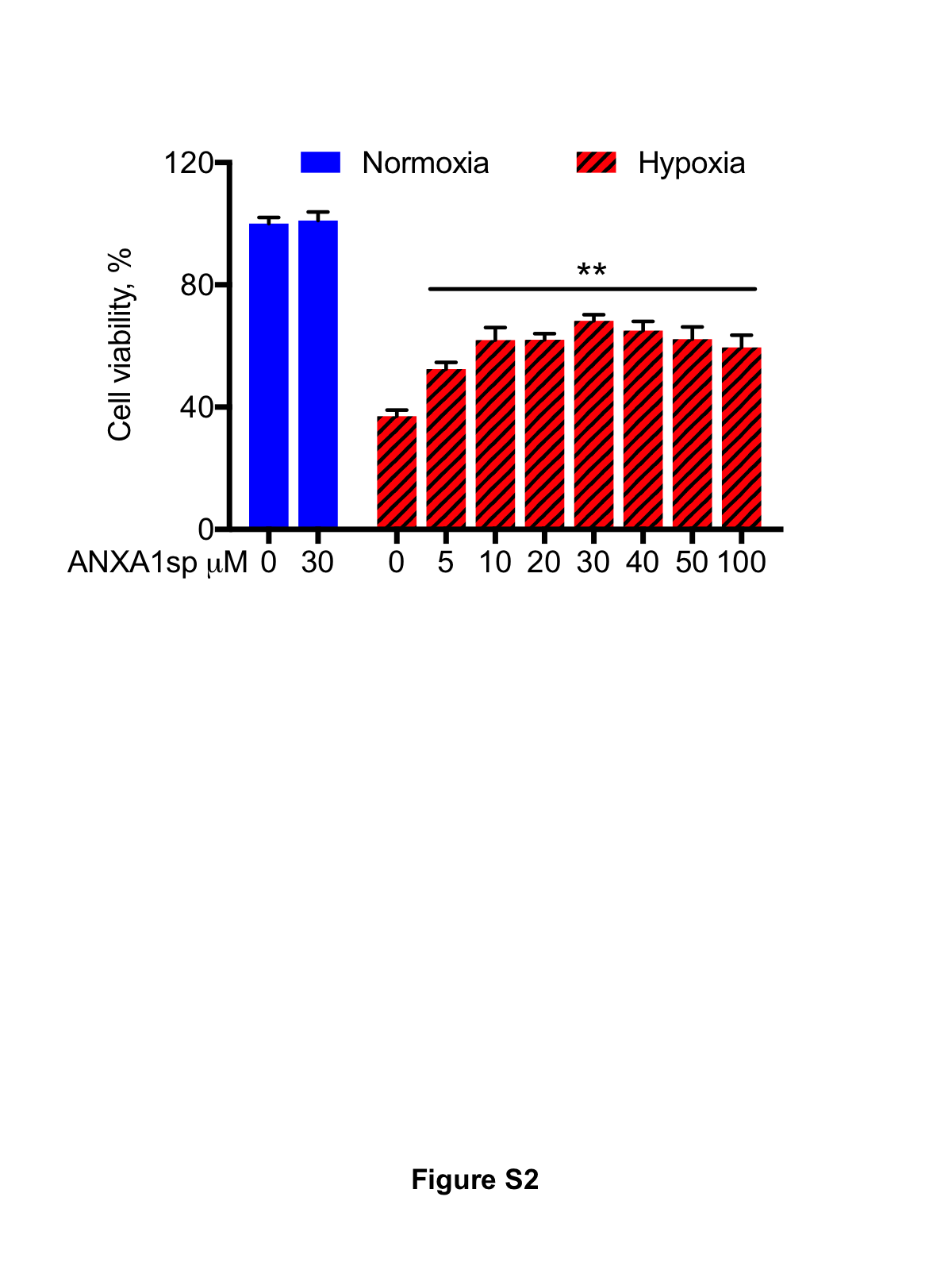

Supplement: Figure S2 — Cell viability measured by MTT assay at 24 h OGD/reoxygenation. [file Image_2.TIFF]
